# An inferior competitor is a successful invader due to its stress tolerance and productivity

**DOI:** 10.1038/s41598-023-48152-y

**Published:** 2023-11-24

**Authors:** Yohannes B. Tesfay, Annika Blaschke, Juergen Kreyling

**Affiliations:** Experimental Plant Ecology, Institute of Botany and Landscape Ecology, University of Greifswald, 17489 Greifswald, Germany

**Keywords:** Plant ecology, Invasive species

## Abstract

The invasion of ecosystems by non-native species is recognized as one of the most significant global challenges, particularly in semiarid regions where native biodiversity is already under stress from drought and land degradation. The implicit assumption is that invaders are strong competitors, but a greenhouse pairwise experiment conducted to examine intraspecific and interspecific competition effects of *Opuntia ficus-indica*, a widespread invader in semiarid ecosystems, with two species native to the highlands of Eritrea, *Ricinus communis* and *Solanum marginatum*, revealed that *O. ficus-indica* is a weak competitor. The unique ability of *O. ficus-indica*’s fallen cladodes to undergo vegetative growth becomes a fundamental trait contributing to its spread. This growth strategy allows *O. ficus-indica* to outgrow native species and establish a significant presence. In direct interaction, the competition in aboveground productivity measured by the logarithmic response ratio for *O. ficus-indica* was 3.4-fold and 5.9-fold higher than for *R. communis* and *S. marginatum*, respectively. Belowground, the native *R. communis* was facilitated (− 1.00 ± 0.69) by *O. ficus-indica* which itself suffered from high competition. This pattern became even more evident under water shortage, where aboveground competition for *S. marginatum* decreased 5.7-fold, and for *O. ficus-indica*, it increased 1.4-fold. Despite being a poor competitor, *O. ficus-indica* outperformed *R. communis* and *S. marginatum* in both aboveground (4.3 and 3.8 times more) and belowground (27 and 2.8 times more) biomass production, respectively. The findings of this study challenge the common interpretation that invasive species are strong competitors and highlight the importance of considering other factors, such as productivity and tolerance limits when assessing the potential impacts of invasive species on semiarid ecosystems.

## Introduction

Invasive plants are often found to outcompete native species during their successful invasions of many ecosystems^1^. Thereby, invasive species can endanger populations of native species^2–4^, reduce spatial diversity^5^, or negatively affect crop yields^6^. All of these processes are commonly explained by the superior competitive abilities of the invasive species^7^. Moreover, the ability of the invasive species to adapt to a new environment can be influenced, either positively or negatively, by the presence of other species^8^. Some argue that only highly competitive species can spread in a new environment^9^ after successfully overcoming biotic and abiotic barriers^10^.

Field observations show that invasive species outcompete and limit the abundance of native species^7,11,12^ and direct paired competition experiments generally support this finding^7^. Exceptions with highly competitive native species limiting the exotic invaders mainly stem from temperate grasslands^7^. Here, competition by native species has been shown to reduce invasive plant growth considerably and has an even stronger effect than herbivory. Accordingly, the high competitive abilities of native species can effectively reduce invasion^13–15^. Although some communities or ecosystems are more susceptible to invasions than others, there is limited understanding of the competitive balance between invaders and native species in subtropical semiarid shrublands^7^, where unpredictable precipitation can cause strong abiotic stress as well as temporarily abundant available resources.

The susceptibility of communities to invasion is often increased by the presence of available resources due to fluctuating environments in time or disturbances in space^16,17^. The probability of an invasion occurring is closely linked to the resource availability during the invasion period, and this availability is, in turn, impacted by the degree of disturbance in that particular ecosystem^16^. Consequently, ecosystems in unpredictable climates where a limiting resource, here water, creates chronic stress but intermittent rainfall events causing unlimited availability at hardly predictable points in time, can be expected to offer little resistance to invasions. Subtropical semiarid drylands are such ecosystems, but little is known about invasion processes and competitive balances between invaders and native species in these ecosystems^7^.

Another factor that plays an important role in invasion success is stress tolerance, the ability of a species to withstand and cope with various abiotic and biotic stress factors, such as drought, temperature extremes, or competition for resources^18^. As a result, this tolerance has the potential to further affect the competitive balances, particularly in resource-limited ecosystems such as the water-limited semi-arid subtropical shrublands. In the context of plant competition, water availability plays an important role in affecting the level of competition and the mechanisms plants use to compete for resources^19^. For instance, limitation of available water may cause plants to increase their root growth to access more water, which can eventually intensify competition for water and nutrients in the soil^20^. Additionally, water stress can impair plant growth and increase the proportion of visibly wilted leaves, affecting the overall health and competitive ability of the plants^21,22^. The highly water-efficient CAM (Crassulacean Acid Metabolism) photosynthetic strategy could benefit invasive species in such an environment as a so-called ‘novel weapon’^23,24^.

*Opuntia ficus-indica* (prickly pear) is a cactus native to Mexico which is exceptionally successful in invading arid and semi-arid ecosystems, e.g., in Australia, South Africa, Kenya, Tanzania, Ethiopia, Eritrea, Somalia, Yemen, North America, and Hawaii^25–27^. Moreover, it is reported that *O. ficus-indica* is the most widespread invasive cactus, which has been found nowadays in 22 different countries on all continents except Antarctica outside its native range^28^. It is reported to alter the composition of the indigenous plant and animal communities^29^ and to reduce spatial diversity^5^. Its invasion has economic effects by impeding the movement of livestock and humans, as it forms impenetrable thickets and thereby threatens large-scale cattle ranching^26^. The spiny nature of *O. ficus-indica* impedes browsing and grazing, and in heavily infested areas, livestock struggle to access grazing areas. This negatively impacts cattle ranching, reducing livestock numbers and harming the local economy^30,31^. Despite studies recognizing the utilization and grazing barriers of the cactus^32,33^, we are unaware of clear data on the extent of the economic effects. Furthermore, *O. ficus-indica* capitalizes on its important high water use efficiency as a CAM plant. This adaptation not only grants drought tolerance but also empowers the plant to thrive in arid conditions, enabling survival with minimal precipitation^34,35^.

Based on its invasion success, *O. ficus-indica* is assumed to outcompete the neighbouring native species^36^ and, due to its strong drought tolerance^37,38^, can become even more competitive when water availability is scarce. Coincidentally, projections of precipitation in vast parts of the areas invaded by *O. ficus-indica*, with ongoing climate change, will face increasing droughts in future, leading to water scarcity^39^. The competitive abilities of *O. ficus-indica*, however, have not yet been tested experimentally, neither under water-limited nor under wetter conditions.

Accordingly, this paper deals with pairwise competition experiments in two different water availabilities between the invasive plant *O. ficus-indica* and two typical and common native plant species of invaded areas in Eritrea, *Ricinus communis* and *Solanum marginatum*. Considering its global invasion success in dry ecosystems, we hypothesized that *O. ficus-indica* has a high competitive power and outcompetes the native species irrespective of water availability. We furthermore expected that its competitive superiority increases under water stress.

## Materials and methods

Competitive balance between the invasive *O. ficus-indica* and two species native to the highlands of Eritrea, *Ricinus communis* and *Solanum marginatum*, was experimentally evaluated. All three species grow together in the highlands of Eritrea^5^ at altitudes above 1500 m with a mean annual rainfall of 500 mm. It is an area with a warm to cool semiarid climate and potential evapotranspiration ranging between 1300 and 1800 mm. In this area, the rainy season normally lasts about three months, beginning in June and ending in August. Besides heavy rain, occasional showers come in March and April^5,46^. All three plant species/ seeds were collected from the highlands of Eritrea in 2018 with the permission obtained from the Ministry of Agriculture, Regulatory Services Department with a certificate issue number ER-PSC-00026. The species were identified following Hedberg and Edwards^47^, Edwards et al.^48–50^, Hedberg et al.^51,52^ Mesfin^53^, Bein et al.^54^, and by comparing the collected specimens at the Herbarium of the Eritrea Institute of Technology. The plant collection and use were in accordance with all the relevant guidelines.

### Study species

Vilà et al.^6^ and Vilà and Weiner^7^ criticise pairwise competition experiments between invasive and native species for selecting highly competitive and aggressive invaders and comparing them to rare and threatened native species, which, per se, are poor competitors. Here, we avoid this bias by comparing a globally successful invasive species to two common and widespread native species that overlap in range and are known to be tolerant to disturbance.

*Opuntia ficus-indica* (L.) Mill. (Cactaceae) is an evergreen perennial plant that can grow up to 5 m in height. The species has succulent stems that are formed as a sequence of flattened segments, the cladodes, which generally have elliptical bases that supports the greatly enlarged, flattened upper portions. *O. ficus-indica* has spines, morphologically corresponding to leaves. Its flowers (5–10 cm in diameter) are sessile and solitary, and the fruits are berries that are 4–8 cm in diameter^55,56^. Nieddu and Chessa^56^ found the germination of *O. ficus-indica* seeds reaching up to 90% in growth chambers with a day/night temperature of 30/20 °C, but only reaching 55% when seeds were kept at room temperature and 43% when seeds were placed outdoors. The seeds are usually dispersed after the consumption of the fruits by humans, birds, and other animals (endozoochory). The seeds require comparatively long time for germination due to their hard, lignified integuments which need to be overcome by physical or chemical reactions^57^. Furthermore, vegetative propagation occurs through cladodes readily taking root upon falling to the ground and conspicuous patch formation is an important factor in the persistence of local populations of the plant, although seedling recruitment is essential for expanding the geographic range and establishment in new areas^55^.

*Ricinus communis* L. (Euphorbiaceae) is a fast-growing, soft woody shrub or small tree (up to 5 m tall) and utilizes the C3 photosynthetic pathway. *R*. *communis* is indigenous to eastern Africa, the south-eastern Mediterranean Basin, and India, and it is commonly distributed throughout the tropics and warm temperate regions. It has developed various strategies, such as rapid growth, allelopathy, thriving in a wide range of soil conditions, and high seed production, to adapt to the conditions of disturbed areas^58^. *R. communis* is also known as a poisonous plant due to the presence of toxic ricin and ricinine in its seeds and other parts, however, *R*. *communis* is still commonly used as an ornamental plant and for its antimicrobial features, it is used as a medicinal plant to treat several ailments^59^.

*Solanum marginatum* L. (Solanaceae), native to the highlands of Eritrea and Ethiopia, is a perennial shrub that follows the C3 photosynthetic pathway. It can grow up to 2 m tall and its leaves are densely covered in white stellate hairs and armed on both the upper and lower surfaces with prickles^60^. In its native range, it usually occurs in disturbed areas between altitudes of 2000 m and 3000 m above sea level^61,62^. *S*. *marginatum* is usually unpalatable to herbivores mainly due to the presence of a poisonous alkaloid chemical compound^63,64^.

These three species have all been referred to as weedy, thrive in disturbed areas^58,62,65^, and possess chemical and/or physical defences against herbivory. They differ little in their potential plant heights, but in some morphological features; *O. ficus-indica*, as a cactus is a succulent plant that can store water, unlike the other two native species. Sharing a similar life strategy and avoiding the common bias of choosing rare native species^6,7^ were the important considerations for choosing these two native species.

### Experimental design

The competition between species can be quantified experimentally using indices based on pairwise experiments which express competition intensity, effects, and response^3,40^. Competition indices help to quantify the proportional decrease in native plant performance due to the competing effects of invasive species and compare the effects on different species or under different environmental conditions^40–43^. When interspecific competition is weaker than the intraspecific competition in an invaded ecosystem, each native species in that community limits its own population growth more than it limits that of the competitive invader^44,45^.

The greenhouse competition experiment was carried out from February 2020 to May 2021 at Greifswald, Germany. Two species native to the highlands of Eritrea, *Ricinus communis* and *Solanum marginatum*, were selected to test the competitive potential of the invasive cactus, *O. ficus-indica* in a common-garden pairwise competition experiment. Two different treatments were set up based on resource availability (water), depicting dry and wet environments. Each treatment was prepared with fifty pots with a volume of one litre. The substrate was a mixture of 75% loamy forest soil and 25% quartz sand. All plants were raised from seeds. Since *O. ficus-indica* needed more time to germinate, it was sown in January 2019, eight months ahead of the other two plants, which were sown in August 2019. After being transplanted into their respective target pots on February 10th, 2020, all plants, regardless of the species, exhibited similar heights of approximately 15 cm and no conspicuous difference in belowground biomass (personal observation). The plants were categorized into monocultures of each species (intraspecific competition) and polycultures of each native together with the invasive (interspecific competition). The intraspecific category had a pair of *R. communis* plants per pot (20 replicates), a pair of *S. marginatum* plants per pot (20 replicates), and a pair of *O. ficus-indica* plants per pot (20 replicates). The interspecific category had *O. ficus-indica* and *R. communis* plants per pot (20 replicates) and *O. ficus-indica* and *S. marginatum* plants per pot (20 replicates). Half of the pots from each category were distributed to each condition of the wet and dry environment. The pots in the wet environment were watered twice per week with 100 ml of water, simulating a wet condition, the other half of the pots were watered only once per week with 100 ml. The watering regimes were based on pre-trials with all three species. The dry variant was set right above the limit at which the native species showed strongly increased mortality. The invader *O. ficus-indica* proved to be remarkably resilient, surviving for nine months without any watering. To assess the plant's water tolerance limits, we conducted a pot experiment, exposing 16 plants to a gradient of water availability ranging from no water up to 260 ml twice a week over a period of nine months. While the growth of the plants exposed to drought on the lower end of the gradient was impeded, all plants survived the experiment, even the one not receiving any water for 9 months. In a subsequent recovery experiment, the plants that were previously subjected to drought showed rapid recovery within 5 days. Additionally, the plant that received the highest amount of water was submerged in a bucket of water for three months and displayed no signs of stress; instead, its roots grew upward and out of the water (see Supplementary 1 online for details on the pretrial).

The effects of the invader in the main experiment were assessed by comparing the native species growing alone (intraspecific, i.e., the average of the two plants per pot) with those that were growing with the invader (interspecific) for the wet and dry treatment, respectively. The positions of the pots within the greenhouse were frequently interchanged to ensure similar environmental conditions and reduce edge effects of the glasshouse or general heterogeneity of environmental conditions. The plants were kept at an average of 40% humidity and in a 12-h day and night cycle, at temperatures of 20 °C and 12 °C, respectively.

### Response parameters

We quantified above- and belowground net primary production at the end of the experiment (after 15 months of growth in competition), hereafter ANPP (Aboveground Net Primary Productivity) and BNPP (Belowground Net Primary Productivity). Belowground biomass was gently washed free from the substrate by rinsing it into a coarse sieve so that the substrate was washed away, and the roots and rootlets could all be collected. The above- and belowground biomasses were dried for five days at 60 °C and 100% ventilation and weighed.

### Statistical analyses

The growth parameters (ANPP and BNPP) were analysed using a two-factorial analysis of variance (ANOVA^66^) with the explanatory factors being water regime (wet/dry) and competition (intraspecific/interspecific for both the native species and the invader, i.e., a factorial variable with four levels: native–native, native–invasive, invasive–native, and invasive–invasive, with the biomass value of the first named in each pair in interspecific competition and the average biomass of both individuals per pot in case of intraspecific competition) including their interaction. Single models were run for each native species (*S. marginatum* and *R. communis*) and each response parameter (ANPP and BNPP) for a total of four ANOVA analyses. Tukey’s HSD post hoc tests^67^ were used to assess the significance of differences in pairwise comparisons for significant interaction terms. Similarly, the same representation based on the total biomass production is also provided in the Supplementary 2 online.

Furthermore, two different competition indices were implemented; Logarithmic Response Ratio (lnRR) and Relative Neighbour Effect (RNE)^3,68,69^. As both indices yielded highly similar patterns, we present the results based on lnRR in the main text (Fig. 2) and based on RNE in the Supplementary 2 online.

The Logarithmic Response Ratio (lnRR) is computed by the natural log of the ratio between the mean value of the respective control treatment (intraspecific without a second species) and the value of each treatment growing in competition with a second species (interspecific). Smaller values indicate weaker competition with negative values showing facilitation, while larger values indicate intense competition between the species^3,43^. lnRR is expressed as:

$$ lnRR = \ln \left( {{\raise0.7ex\hbox{${P_{contr} }$} \!\mathord{\left/ {\vphantom {{P_{contr} } {P_{mix} }}}\right.\kern-0pt} \!\lower0.7ex\hbox{${P_{mix} }$}}} \right) $$

where ln is the natural logarithm, $P_{contr}$ is the performance of the plant growing in a monoculture and $P_{mix}$ is the performance of a plant growing in a mixture.

The effect of the different water regimes on the competition index data was examined using a one-way analysis of variance with water regime (dry/wet) as explanatory factor. Single models were run for each native species (*S. marginatum* and *R. communis*) and each response parameter (ANPP and BNPP) for a total of four ANOVA analyses.

Parametric assumptions were checked for all ANOVA models by examining the diagnostic plots (residuals versus fitted plots for homoscedasticity of the residuals and normal qq-plots for normal distribution of residuals^70^. According to the diagnostic plots, the ANPP and BNPP for both the *R. communis* and the *S. marginatum* datasets were log(x + 1)-transformed. The competition index datasets did not require transformations. For graphical visualizations, the function bar graph.CI in the R package sciplot^71^ was used. All statistical analyses were done in R version 4.2^72^.

## Results

**Figure 1:**
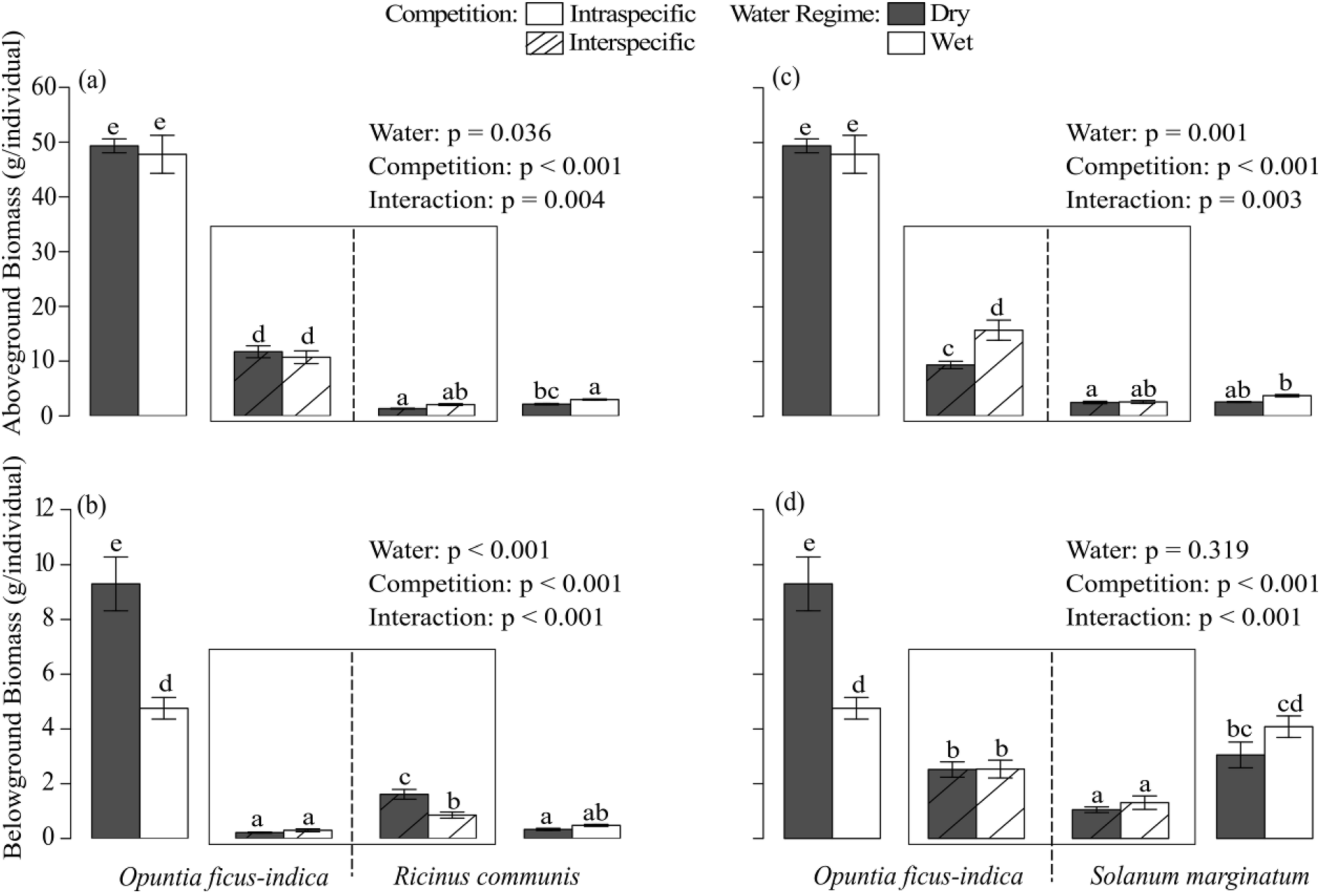
Aboveground (**a**, **b**) and belowground (**c**, **d**) biomass production (mean ± SD) of *Opuntia ficus-indica* and *Ricinus communis* growing in intraspecific competition or interspecific competition (**a**, **c**) and *Opuntia ficus-indica* and *Solanum marginatum* growing in intraspecific competition or interspecific competition (**b**, **d**) under wet (white) and dry (dark grey) conditions. Competition is a factorial variable with four levels: native-native, native-invasive, invasive-native, and invasive-invasive, with the biomass value of the first named in each pair in interspecific competition and the average biomass of both individuals per pot in case of intra-specific competition. Lowercase letters above the columns indicate homogeneous groups according to Tukey’s post hoc test. The interspecific competition is indicated by diagonal hatching and solid boxes around the bars of those plants that grew together.

ANPP of *O. ficus-indica* was 4.3-fold higher under intraspecific competition than when competing with *R. communis* (Fig. 1a). In comparison, the native *R. communis* was 14-fold less productive aboveground than *O. ficus-indica*, but its ANPP was unaffected by the identity of its neighbour as its production did not differ between intra- and interspecific competition. *O. ficus-indica* was unaffected by the water regime in ANPP while the native *R. communis* produced 2.2 times more aboveground biomass under wet conditions and intraspecific competition than under dry conditions and interspecific competition (Fig. 1a).

BNPP of *O. ficus-indica* was 27-fold higher under intraspecific competition than when competing with *R. communis* (Fig. 1b). In comparison, the native *R. communis* was 17-fold less productive belowground under intraspecific competition than *O. ficus-indica*, but its BNPP even increased 3.1 times under interspecific as compared to intraspecific competition. *O. ficus-indica* doubled its BNPP under the dry as compared to the wet water regime when growing with itself but produced very little BNPP and showed no water effect when grown under interspecific competition. *R. communis*, in contrast, increased its BNPP in dry conditions 1.9 times over wet conditions only when growing in interspecific competition with *O. ficus-indica*, but not when growing with itself (Fig. 1b).

ANPP of *O. ficus-indica* was 3.8-fold higher under intraspecific competition than when competing with *S. marginatum* (Fig. 1c). In comparison, the native *S. marginatum* was 11-fold less productive aboveground than *O. ficus-indica*, but its ANPP was unaffected by the identity of its neighbour as its production did not differ between intra- and interspecific competition. In interspecific competition with *S. marginatum, O. ficus-indica* increased its ANPP 1.7-fold under wet as compared to dry conditions, while it did not show an aboveground growth response to the water regime when growing with itself. The ANPP of the native *S. marginatum* was unaffected by the water regime, irrespective of competition (Fig. 1c).

BNPP of *O. ficus-indica* was 2.8-fold higher under intraspecific competition than when competing with *S. marginatum* (Fig. 1d). In comparison, the native *S. marginatum* was only half as productive belowground as *O. ficus-indica* under intraspecific competition but showed a pattern similar to the latter as its BNPP was 3.0-fold higher when grown with itself than under interspecific competition. *O. ficus-indica* doubled its BNPP under the dry as compared to the wet water regime when growing with itself but produced less BNPP and showed no water effect when grown under interspecific competition. *S. marginatum* showed no significant effect in its BNPP on the water treatment (Fig. 1d).

### Competition index

**Figure 2:**
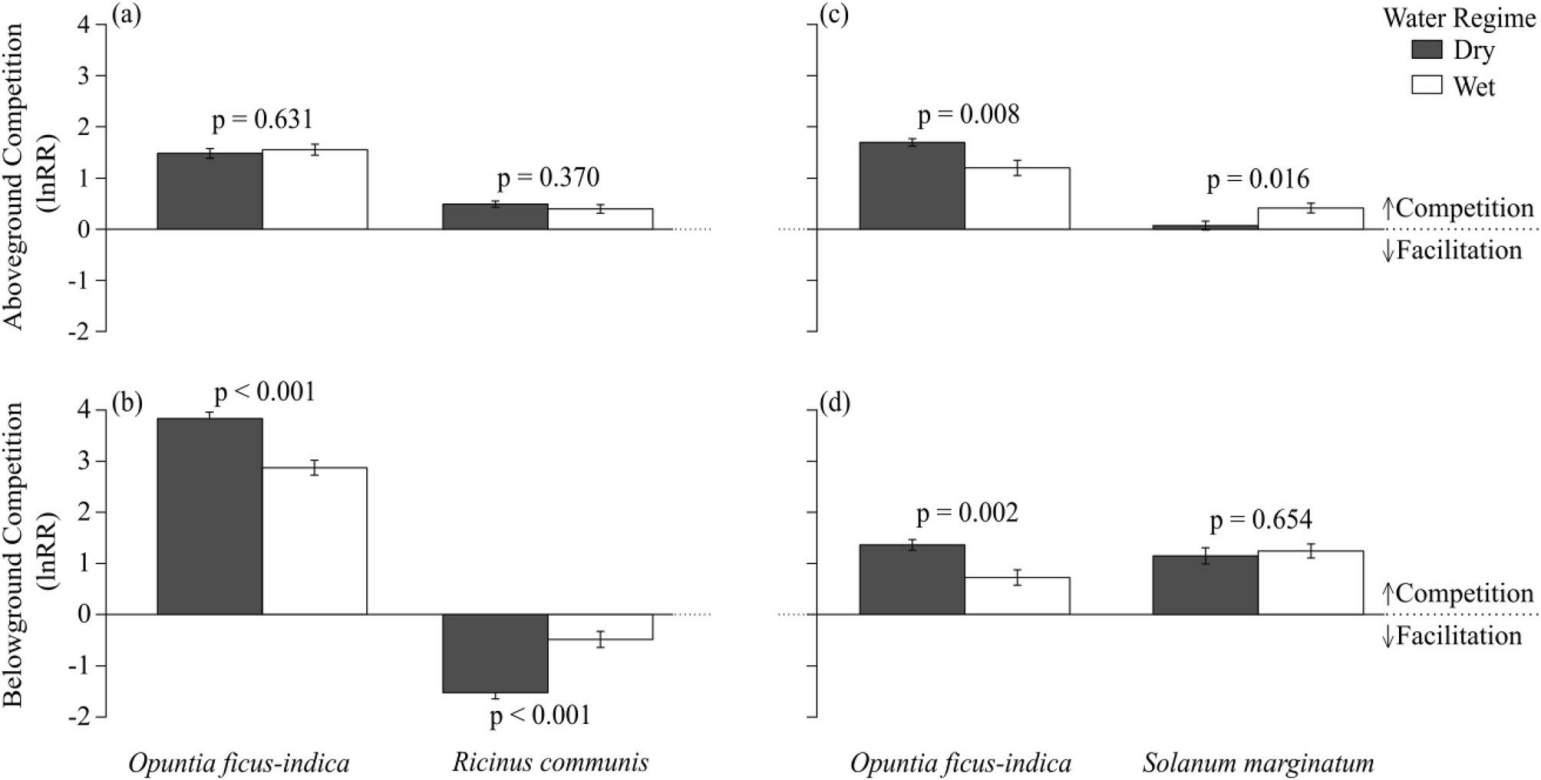
Relative competition intensity, according to the Logarithmic Response Ratio (lnRR) (mean ± SD), of the above (**a**, **b**) and belowground (**a**, **d**) biomass production for the pairwise competition experiment between the invasive *O. ficus-indica* and the native *Ricinus communis* (**a**, **c**) or the native *Solanum marginatum* (**b**, **d**). Negative values indicate a facilitative effect on the named species and positive values indicate competition for the named species. One-way ANOVA pairwise comparisons between the wet (white) and dry (dark grey) water treatments are provided.

*Ricinus communis* and *O. ficus-indica* showed aboveground competition in their direct interaction, with the lnRR for ANPP being 3.4-fold higher for *O. ficus-indica* than for *R. communis* (1.51 ± 0.31 mean ± SD versus 0.45 ± 0.23, respectively; Fig. 2a). This pattern of aboveground competition was unaffected by the water regime. Belowground, the native *R. communis* was facilitated (− 1.00 ± 0.69, Fig. 2b) by *O. ficus-indica* which itself suffered strongly from high competition by the native species (3.35 ± 0.65; Fig. 2b). This pattern was significantly stronger for both species under dry than under wet conditions.

*Solanum marginatum* and *O. ficus-indica* showed aboveground competition in their direct interaction, which was 5.9-fold higher for *O. ficus-indica* than for *S. marginatum* (1.45 ± 0.44 versus 0.24 ± 0.33, respectively; Fig. 2c). This aboveground competition was 5.7-fold stronger for *S. marginatum* under wet as compared to dry conditions and 1.4-fold weaker for *O. ficus-indica* under wet as compared to dry conditions. Belowground, *S. marginatum* and *O. ficus-indica* showed about equal competition in their direct interaction (1.20 ± 0.46 versus 1.04 ± 0.52; Fig. 2d). For *S. marginatum*, belowground competition was unaffected by the water regime while competition increased 1.9-fold under dry as compared to wet conditions for *O. ficus-indica*.

## Discussion

*Opuntia ficus-indica,* a highly successful invader of semiarid and arid ecosystems on all continents containing such conditions^28^, was an inferior competitor to *Ricinus communis* and *Solanum marginatum*, two species native to the highlands of Eritrea where *O. ficus-indica* is also spreading vigorously. These findings stem from our greenhouse pairwise competition experiment offering valuable insights to a possible explanation of potential outcomes in a field setting. *O. ficus-indica* dropped in ANPP and BNPP about fourfold and 3 to 27-fold, respectively, when growing together with *R. communis* and *S. marginatum*. Competition indices indicated high competition for *O. ficus-indica* irrespective of the competing native, while the natives experienced less competition or even facilitation by *O. ficus-indica*. These findings reject the hypothesis that the successful invader is a superior competitor to the native species^73,74^. The majority of pairwise competition trials between invasive and native species supported that hypothesis^7^. The notable exceptions in that meta-analysis stem mainly from temperate grasslands, which are known to harbour native species with high competitive abilities that can resist invasions^75^. Based on this and the competitive abilities found in our study, our invasive species should face strong resistance and have little success in invading native communities containing the two competitively superior native focal species. Surprisingly, that is not the case. *O. ficus-indica* spreads vigorously into areas containing those two native species^5^. So, the question arises: how can an inferior competitor be a successful invader?

The invasive *O. ficus-indica* produced several times more biomass than the natives, even under competition with the natives where it is doing much worse than on its own. Therefore, it ‘outproduces’ the competitively stronger natives in all cases except for BNPP with *R. communis*. Although we didn't quantify belowground biomass at transplantation to keep root systems intact, visual assessment revealed no significant differences among the three plant species. Thus, to better understand the role of competition in plant invasion, the relative biomass production of an invasive species compared to the native species should therefore be taken into consideration. Based on a meta-analysis of interspecific and intraspecific competition trials, Vilà and Weiner^7^ found that mixtures of native and invasive species are less productive than native monocultures but not less productive than monocultures of the invasive plants. In our study, the interspecific mixtures produced, on average, 13.9 g aboveground biomass per pot, which is more than twice the production of the native species in monoculture (5.7 g) but nearly seven times less than the invasive species in monoculture (97.1 g). The numbers for BNPP followed a similar pattern, being 2.9 g for the mixture, 3.3 g for the native monocultures, and 14.1 g for the invasive monocultures, respectively. Comparing these numbers to the meta-analysis implies that our finding of a highly productive but weakly competitive invasive species might be an interesting exception rather than the rule.

The results of our experiment showed that competition by the native species generally increased for *O. ficus-indica* under the dry as compared to the wet treatment (Fig. 2). At first sight, this finding contradicts our second hypothesis which expected higher competitive power of *O. ficus-indica* under dry conditions. However, this weak competition should not be interpreted as a sign of reduced invasion pressure on the community. *O. ficus-indica* is a plant that has adapted to endure extreme environmental conditions and flourish in arid environments. By utilizing Crassulacean Acid Metabolism (CAM) photosynthesis, *O. ficus-indica* strategically minimizes moisture loss and enhances water-use efficiency, making it a highly drought-tolerant species that can cause pressure on the native community^76^. Moreover, the unique ability of *O. ficus-indica*'s fallen cladodes to undergo vegetative growth is a fundamental trait that contributes to its spread. This growth strategy enables *O. ficus-indica* to outgrow native species and establish a significant presence.

We based the water regimes of our experiment on pretrials that tested the water tolerance limits of the target species and found increased mortality of the native species right below our dry water regime. The ability of *O. ficus-indica* to survive well below this limit probably contributes greatly to its invasion success in dry ecosystems. Moreover, within the dry ecosystems, water availability becomes a major source of competition, favouring plants like *O. ficus-indica* which are capable of enduring the water shortages^19^, but readily taking up large amounts of water as soon as it becomes available with its rapidly forming rain roots and storage in its succulent tissue^77^. Our pretrial showed that it even survived nine months without any water addition in our experimental setup (see Supplementary 1 online). This extreme drought tolerance is an important advantage for *O. ficus-indica,* as drought is frequent in the highlands of Eritrea^78^. The native species are expected to be adapted to what was the norm frequency and magnitude of drought, but climate change will result in stronger and more frequent drought events^39^, which will challenge their survival. Disturbance in the native vegetation due to drought might therefore create gaps with little to no interspecific competition in which the invasive species can spread even more rapidly^17^. Notably, *O. ficus-indica* was also able to survive very wet periods according to our pretrials. Even complete submergence of the pots for three months did not kill the plants but resulted in roots growing on the surface of the water, probably seeking oxygen access (see Supplementary 1 online). Besides ‘outproducing’ native species by high biomass production, these extreme tolerance limits are another potential explanation for the global invasion success of *O. ficus-indica*.

The two native species showed interesting differences in their response to competition by *O. ficus-indica*. *R. communis* increased its BNPP under interspecific competition with *O. ficus-indica* when stressed with water shortage (dry water regime). This is reflected in the competition index indicating facilitation for *R. communis* by *O. ficus-indica* (Fig. 2). Fighting against *O. ficus-indica* belowground, however, did not help its general case as it was still ‘outproduced’ by *O. ficus-indica* aboveground. The ANPP of *O. ficus-indica* in interspecific competition with *R. communis* is quite remarkable when taking the very low BNPP due to high interspecific competition into consideration. *S. marginatum*, on the other hand, appeared to give in to the interspecific competition by *O. ficus-indica* belowground, as it showed decreased BNPP compared to the intraspecific interaction. *O. ficus-indica* is able to produce about 10 times as much root biomass in the presence of *S. marginatum* than in the presence of *R. communis*. Still, *S. marginatum* was successful in competing with *O. ficus-indica* aboveground, where it showed low competition, especially under dry conditions. Despite the efforts to compete with *O. ficus-indica* aboveground, it did not alter the overall outcome, as it was still ‘outproduced’ by *O. ficus-indica* aboveground. Potential explanations for the different behaviour of the native species in response to competition by *O. ficus-indica* might be that *S. marginatum* unlike *R. communis* is rather a drought-tolerant species that can better survive longer periods without water^62^. In any case, neither strategy appeared to be successful as both natives are clearly outperformed by *O. ficus-indica* in ANPP, irrespective of the water regime and despite them being superior competitors to *O. ficus-indica*.

## Conclusion

A species successfully invading semiarid to arid ecosystems across the globe, *O. ficus-indica*, is an inferior competitor to two common and widespread native species of the highlands of Eritrea. One of the natives, *R. communis*, successfully competed with *O. ficus-indica* belowground, and the other native, *S. marginatum*, showed superior competition mainly aboveground. This finding contradicts the common implicit interpretation that successful invasive species have overall advantageous traits that make them stronger competitors than native species. Being vastly more productive, even under interspecific competition, and considerably more tolerant against water stress than the native species appears more important than competitive power, at least for invasions in semiarid to arid, open vegetation. Stronger and more frequent disturbance of the native vegetation by drought due to climate change will further accelerate the success of *O. ficus-indica*, as it is extremely drought tolerant and produces considerably more biomass in the absence of interspecific competition.

### Supplementary Information

Supplementary Information.

## Data Availability

The datasets generated during and/or analysed during the current study are available from the corresponding author upon reasonable request.
