# Supplementary material for: An inferior competitor is a successful invader due to its stress tolerance and productivity
Source: Sci Rep. 2023 Nov 24;13:20694. doi: 10.1038/s41598-023-48152-y (PMC10673848; doi:10.1038/s41598-023-48152-y)
Supplement: Supplementary file 1 — Supplementary Information. [file 41598_2023_48152_MOESM1_ESM.pdf]

An inferior competitor is a successful invader due to its stress tolerance and productivity

Journal: Scientific Reports

Yohannes B. Tesfay, Annika Blaschke, Juergen Kreyling

Yohannes B. Tesfay*, Annika Blaschke, Juergen Kreyling
Experimental Plant Ecology, Institute of Botany and Landscape Ecology,
University of Greifswald, 17489 Greifswald, Germany

*Corresponding author e-mail: yohannes.tesfay@stud.uni-greifswald.de; yohanntesfay@gmail.com

Supplementary materials

Supplementary 1

Extreme water tolerance limits of the globally invasive cactus *Opuntia ficus-indica*

The tolerance limits of *Opuntia ficus-indica* regarding water supply were examined by monitoring growth (Supplementary Fig. 1 a), biomass production (Supplementary Fig. 1 b and d), water content (Supplementary Fig. 1 c), and soil water potential (Supplementary Fig. 2) in a pot experiment in the same greenhouse and the same experimental settings such as pot size, substrate, plant material, and environmental conditions as the main experiment described in the main paper. Sixteen plants were subjected to an irrigation gradient (0 to 260 ml of water per week) for nine months, four were then observed in a recovery experiment for another three months. After the drought and recovery experiment, the plants' biomass and water content were determined. All plants survived, even though the one subjected to complete drought throughout the trial did not grow. All other plants also showed growth, which was reduced up to a water addition of 60 ml per week and did not further change above that limit. The plants that were previously subjected to drought also showed rapid signs of recovery within five days of the start of the recovery experiment (Supplementary Fig. 4). Concerning drought tolerance, *O. ficus-indica* can survive droughts that last up to nine months and recover quickly. Surprisingly, no sign of reduced performance was found towards the wet end of the gradient and *O. ficus-indica* even survived three months of flooded conditions and produced roots at the top of the water when flooded (Supplementary Fig 3 b). We conclude that *O. ficus-indica* has extremely broad tolerance limits regarding water supply, potentially allowing it to thrive in many regions of the world.

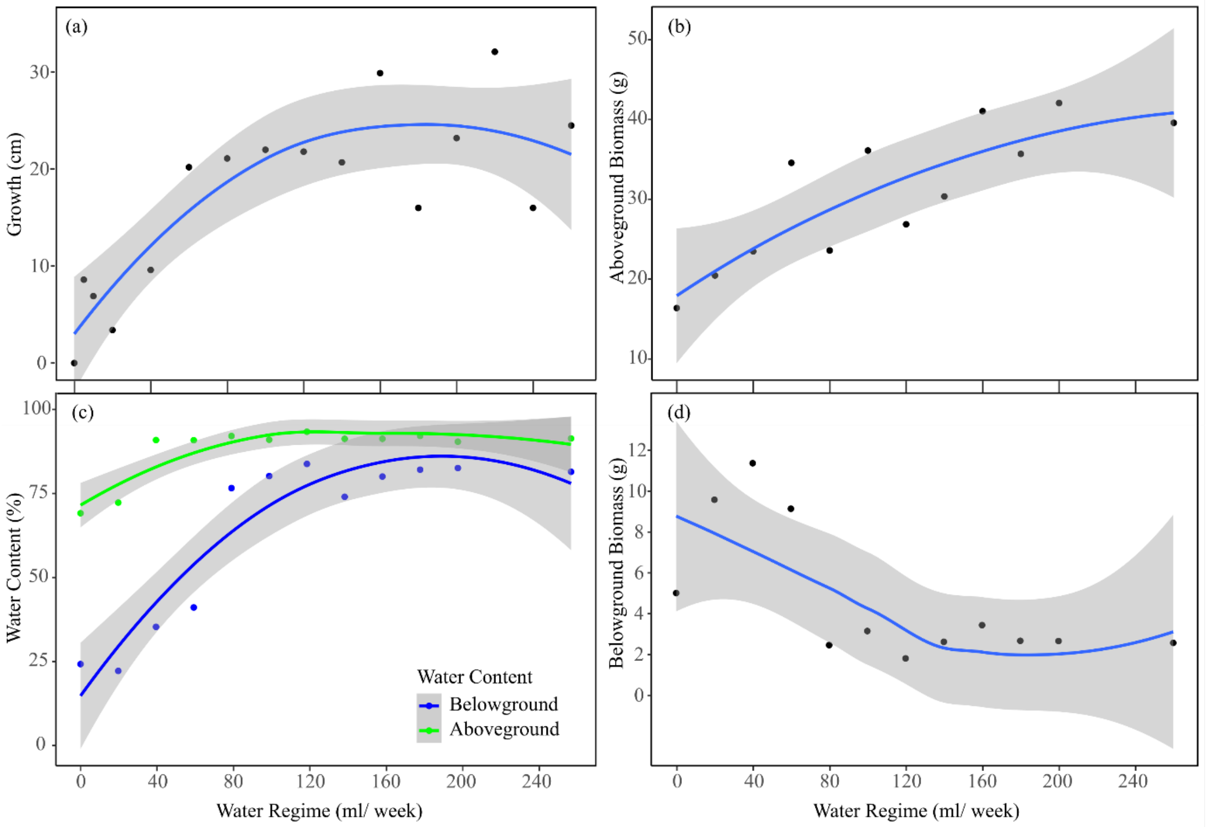

Supplementary Figure 1 Growth (a), aboveground biomass (b), water content (c) and belowground biomass (d) at the end of the drought experiment. Dots are original data points, and lines show the smoothed local polynomial regression fitting (loess). 95% confidence intervals are displayed in grey. This pretrial aimed at observing potentially non-linear patterns of response variables along the water availability gradient. A powerful tool to answer this kind of question is gradient experiments which maximize treatment levels by minimizing replication[1]. To unravel these response patterns, a graphical analysis was performed. Each response parameter was plotted over the water gradient and smoothed conditional means were calculated by Local Polynomial Regression Fitting using the 'loess' function implemented in R[2]. Span was adjusted to produce smooth curves without multiple local extrema based on the assumption that multiple maxima are unlikely in an autecological setting without competition. Confidence intervals (CI) displayed around the conditional means were used to assess the significance of effects at a level of $\alpha = 0.05$. The environmental gradient was considered to have a significant effect if a straight horizontal line could not be fitted inside the 95% CI[3].

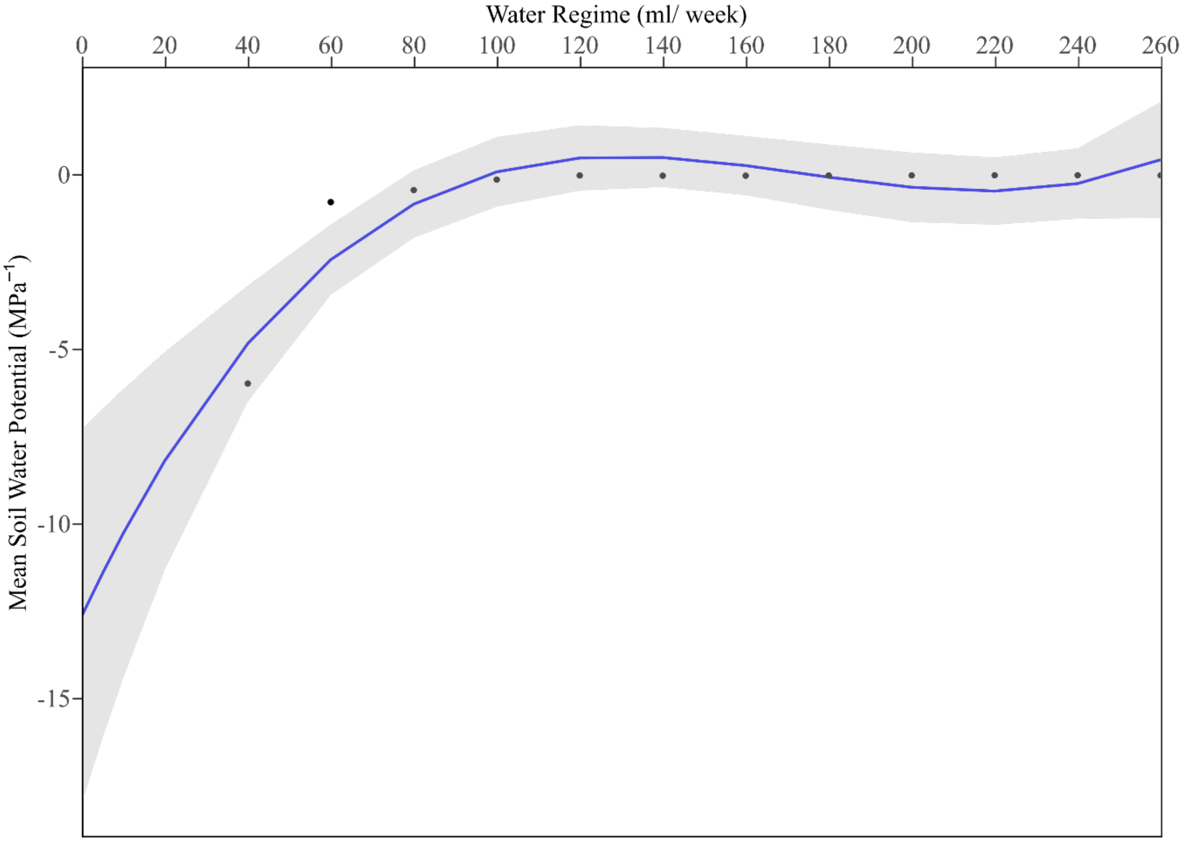

Supplementary Figure 2 Mean water potential calculated from the entire period of drought experiment. Dots are the mean data points, and lines show the smoothed local polynomial regression fitting (loess). 95% confidence intervals are displayed in grey. The sensors used to measure the soil water potential (SWP) had different minimum and maximum limits. We were not aware of the limitations when the sensors were placed in the pots for the experiment. The values of the plants from both the extreme sides of the gradient were influenced by the sensors' lower limit or higher limit, which led to missing data for the driest water treatments p0 to p20, where the SWP dropped below the lower limit of the sensors (Teros 21 generation 1: 2 MPa$_{-1}$). As a result, these plants' SWPs are not included in Supplementary Fig. 2 of the SWP parameter, but we extrapolated the spline so that it reaches 0.

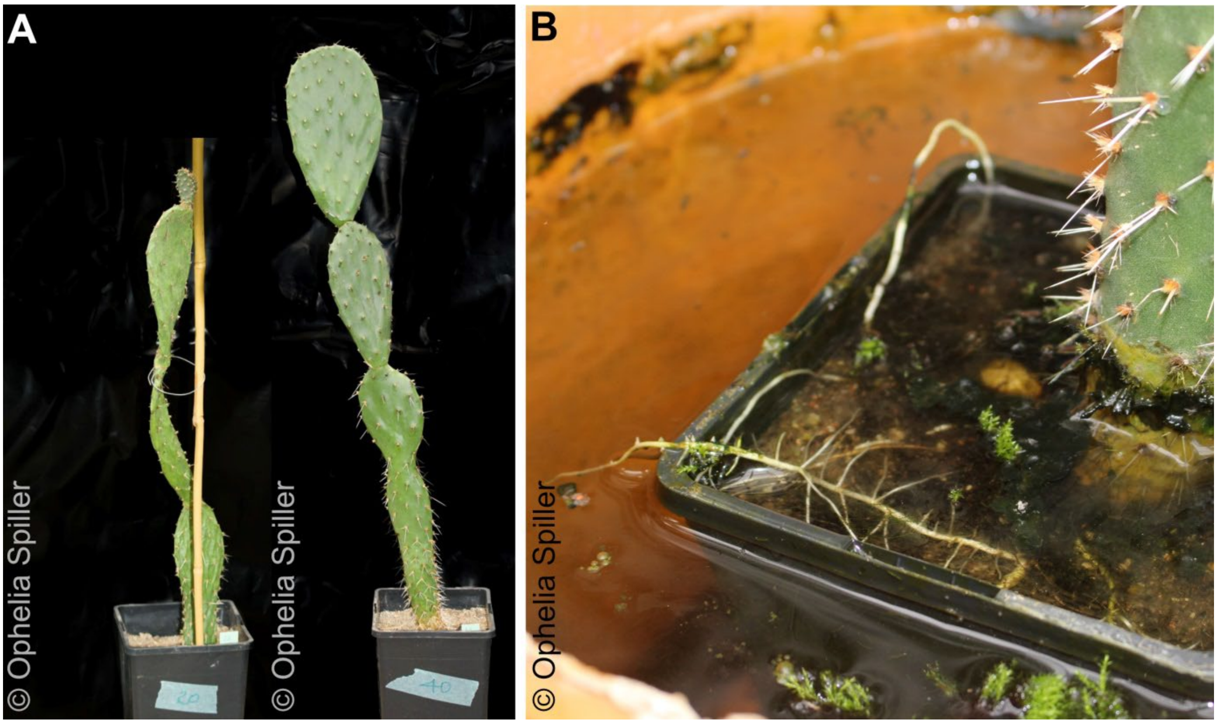

Supplementary Figure 3 Visual observations at the end of the drought pretrial experiment (Day 255). (A) Left: The plant was provided with 20 ml of water twice a week, right: 40 ml twice a week. (B) Close-up photo of the plant that was provided with the highest amount of water throughout the experiment (260 ml twice a week) and later submerged in a bucket full of water for three months. Surprisingly, roots are seen to grow outward on the surface of the water, probably seeking oxygen access.

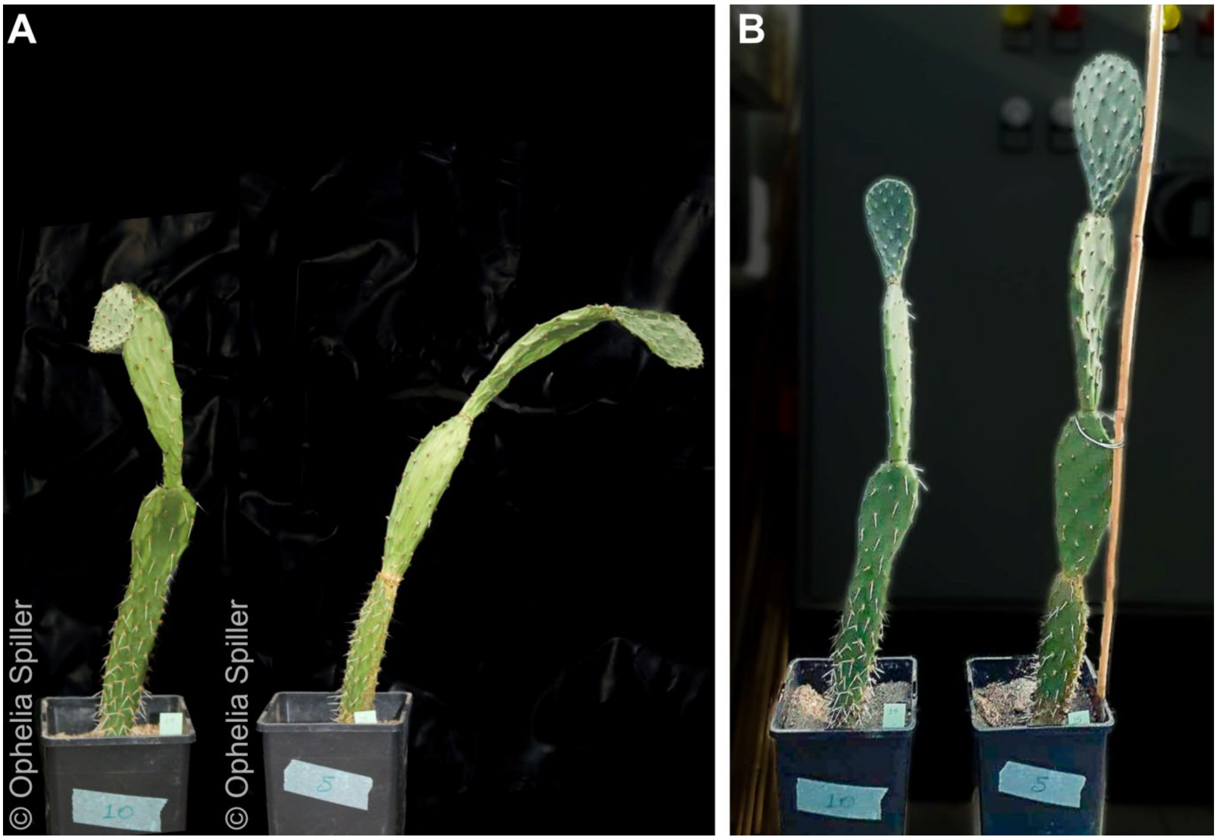

Supplementary Figure 4 (A) Visual observations at the end of the drought pretrial experiment (Day 255). Left: The plant was provided only with 10 ml of water twice a week, right: only 5 ml twice a week. (B) The same plants in a recovery experiment were provided with 100 ml water twice a week. Visual observations within five days of the recovery experiment (Day 260).

Supplementary 2

In Supplementary Fig. 1 and Supplementary Fig. 2 presented here, we illustrate identical results based on the total biomass production of the species.

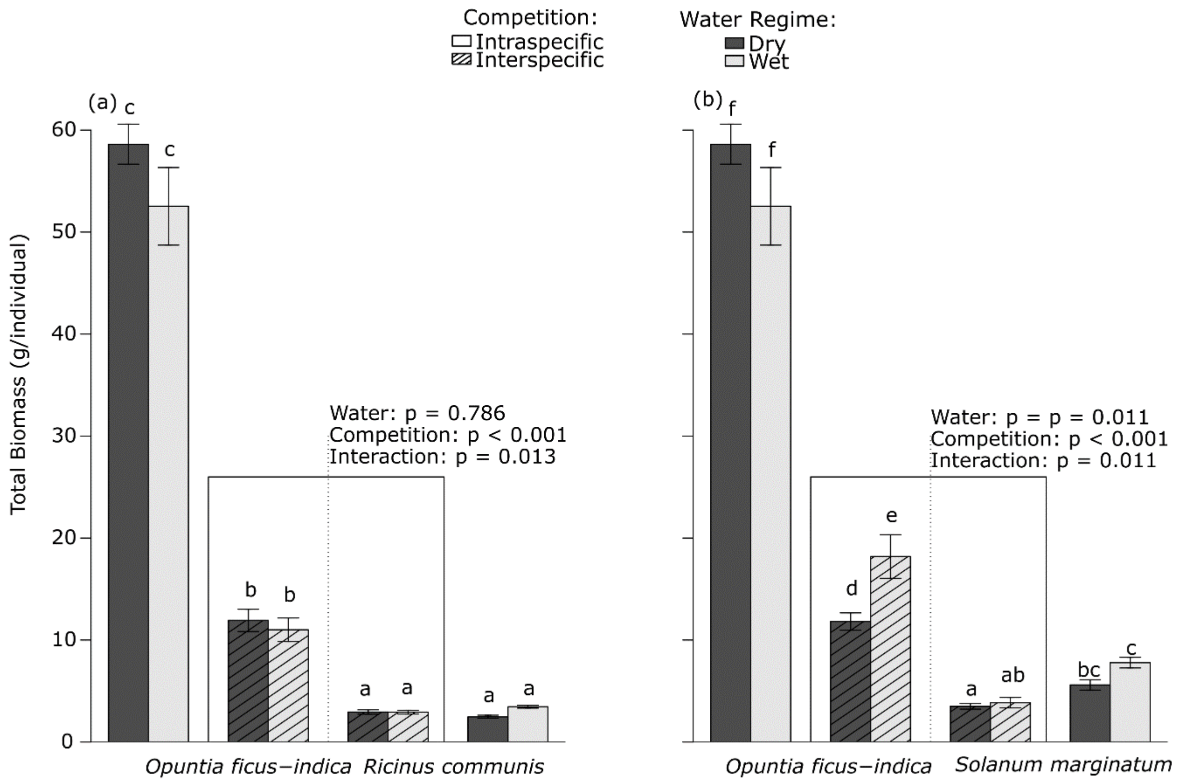

Supplementary Figure 1 Total biomass production (mean ± SD) of *Opuntia ficus-indica* and *Ricinus communis* growing in intraspecific competition or interspecific competition (a) and *Opuntia ficus-indica* and *Solanum marginatum* growing in intraspecific competition or interspecific competition (b) under wet (white) and dry (dark grey) conditions. Competition is a factorial variable with four levels: native-native, native-invasive, invasive-native, and invasive-invasive, with the biomass value of the first named in each pair in interspecific competition and the average biomass of both individuals per pot in case of intra-specific competition. Lowercase letters above the columns indicate homogeneous groups according to Tukey's post hoc test. The interspecific competition is indicated by diagonal hatching and solid boxes around the bars of those plants that grew together.

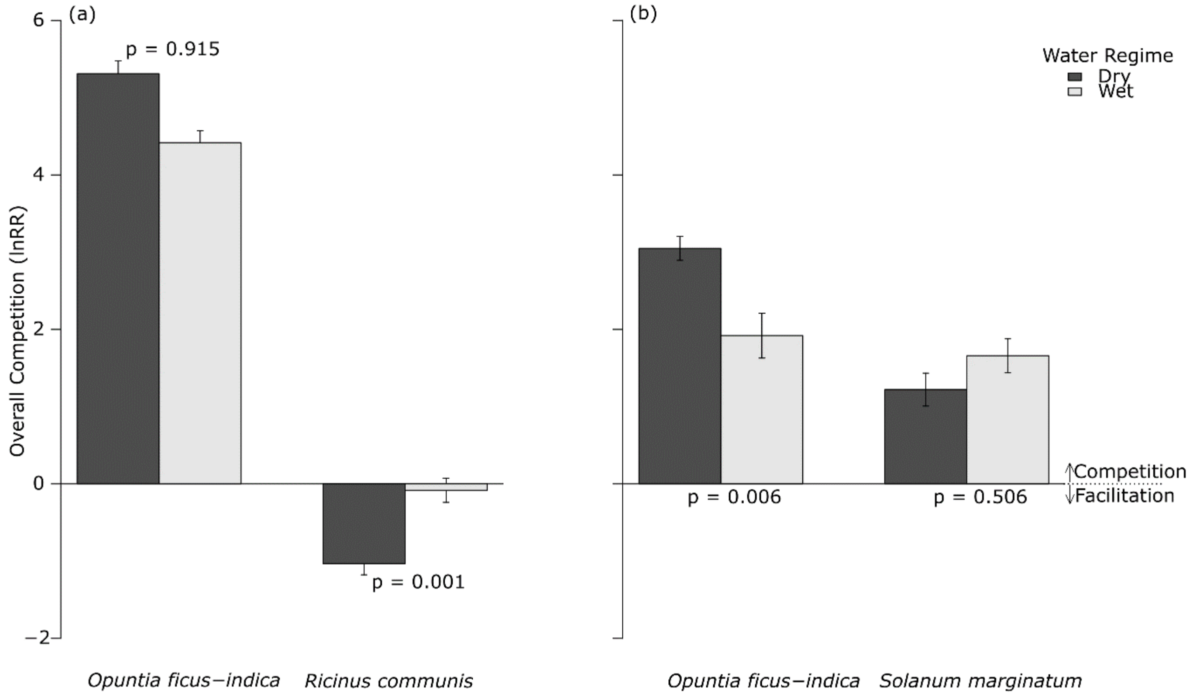

Supplementary Figure 2 Relative competition intensity, according to the Logarithmic Response Ratio (lnRR) (mean ± SD), of the total biomass production for the pairwise competition experiment between the invasive *O. ficus-indica* and the native *Ricinus communis* (a) or the native *Solanum marginatum* (b). Negative values indicate a facilitative effect on the named species and positive values indicate competition for the named species. One-way ANOVA pairwise comparisons between the wet (white) and dry (dark grey) water treatments are provided.

Competition intensity according to the Relative Neighbour Effect (RNE) index.

The Relative Neighbour Effect (RNE) is calculated by subtracting the productivity of a plant growing in a mixed stand with neighboring plants (without a second species or intraspecific) from the productivity of the same plant growing in competition (interspecific) and then dividing this difference by the appropriate denominator, which is either the productivity of the plant growing in competition or the productivity of the plant growing in the mixed stand, depending on which is greater. This index ranges from -1 to 1 with negative values indicating facilitation and positive values indicating competition. RNE is expressed as:

$$RNE = (P_{contr} - P_{mix}) / x$$

$$x = P_{contr} \; if \; P_{contr} > P_{mix}; \; x = P_{mix} \; if \; P_{mix} > P_{contr}$$

P_{contr} is the performance of the plant growing in a monoculture, P_{mix} is the performance of a plant growing in a mixture, and x is P_{contr} when P_{contr} is greater than P_{mix} and x is P_{mix} when P_{contr} is greater than P_{mix}.

The effect of the different water regimes on the competition index data was examined using a one-way analysis of variance with water regime (dry/wet) as an explanatory factor. Single models were run for each native species (*Solanum marginatum* and *Ricinus communis*) and each response parameter (Aboveground biomass-ANPP and Belowground biomass-BNPP) for a total of four ANOVA analyses (Supplementary Fig. 3).

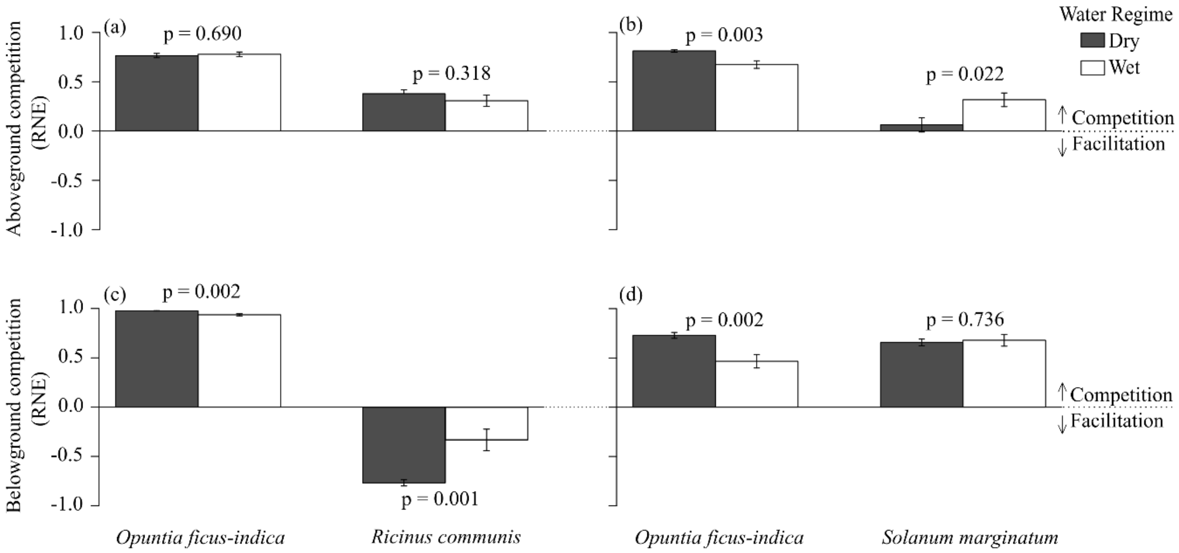

Supplementary Figure 3 Competition intensity according to the Relative Neighbour Effect (RNE) (mean + SE) for above (a, b) and belowground (c, d) biomass production for the pairwise competition experiment between the invasive *Opuntia ficus-indica* and the native *Ricinus communis* (a, c) or the native *Solanum marginatum* (b, d). Negative values indicate a facilitative effect on the named species and positive values indicate competition for the named species. One-way ANOVA pairwise comparisons between the wet (white) and dry (dark grey) water treatments are provided.

References

1. Kreyling, J.; Schweiger, A.H.; Bahn, M.; Ineson, P.; Migliavacca, M.; Morel-Journel, T.; Christiansen, J.R.; Schtickzelle, N.; Larsen, K.S. (2018) To Replicate, or Not to Replicate—That Is the Question: How to Tackle Nonlinear Responses in Ecological Experiments. *Ecol. Lett 21*, 1629–1638. https://doi.org/10.1111/ele.13134.

2. R Core Team (2022) R: A language and environment for statistical computing. R Foundation for Statistical Computing, Vienna, Austria., URL https://www.R-project.org/

3. Gelman, A.; Hill, J. (2007) Data Analysis Using Regression and Multilevel/Hierarchical Models; Analytical Methods for Social Research; Cambridge University Press: Cambridge, UK; New York, NY, USA, 2007; ISBN 978-0-521-86706-1.
